# Dominance of *Haemophilus influenzae* in ear discharge from Indigenous Australian children with acute otitis media with tympanic membrane perforation

**DOI:** 10.1186/1472-6815-13-12

**Published:** 2013-10-08

**Authors:** Heidi C Smith-Vaughan, Michael J Binks, Robyn L Marsh, Mirjam Kaestli, Linda Ward, Kim M Hare, Susan J Pizzutto, Ruth B Thornton, Peter S Morris, Amanda J Leach

**Affiliations:** 1 Menzies School of Health Research, Charles Darwin University, Darwin, Northern Territory, Australia; 2 School of Paediatrics and Child Health, University of Western Australia, Perth, Western Australia, Australia

**Keywords:** Otitis media, *Haemophilus influenzae*, *Moraxella catarrhalis*, *Streptococcus pneumoniae*, Bacterial load, Abundance, Relative abundance, qPCR

## Abstract

**Background:**

Indigenous Australian children living in remote communities experience high rates of acute otitis media with tympanic membrane perforation (AOMwiP). Otitis media in this population is associated with dense nasopharyngeal colonization of three primary otopathogens; *Haemophilus influenzae, Streptococcus pneumoniae* and *Moraxella catarrhalis*. Little is known about the relative abundance of these pathogens during infection. The objective of this study was to estimate the abundance and concordance of otopathogens in ear discharge and paired nasopharyngeal swabs from children with AOMwiP (discharge of not more than 6 weeks’ duration and perforation size <2%).

**Methods:**

Culture and quantitative PCR (qPCR) estimation of *H. influenzae*, *S. pneumoniae*, *M. catarrhalis* and total bacterial load were performed on paired nasopharyngeal and ear discharge swabs from 55 Indigenous children with AOMwiP aged 3.5 – 45.6 months and resident in remote communities.

**Results:**

By culture, *H. influenzae*, *S. pneumoniae*, and *M. catarrhalis* were detected in 80%, 84% and 91% of nasopharyngeal swabs, and 49%, 33% and 4% of ear discharge swabs, respectively. Using qPCR, *H. influenzae*, *S. pneumoniae*, and *M. catarrhalis* were detected in 82%, 82%, and 93% of nasopharyngeal swabs, and 89%, 41% and 18% of ear discharge swabs, respectively. Relative abundance of *H. influenzae* in ear discharge swabs was 0-68% of the total bacterial load (median 2.8%); whereas *S. pneumoniae* and *M. catarrhalis* relative abundances were consistently <2% of the total bacterial load. *S. pneumoniae* and *M. catarrhalis* abundances were significantly lower in ear discharge compared with nasopharyngeal swabs (p = 0.001, p < 0.001); no significant difference was observed in *H. influenzae* mean abundance at the two sites.

**Conclusions:**

*H. influenzae* was the dominant otopathogen detected in ear discharge swabs collected from children with AOMwiP. High prevalence and abundance of *S. pneumoniae* and *M. catarrhalis* in the nasopharynx did not predict ear discharge prevalence and abundances of these pathogens. PCR was substantially more sensitive than culture for ear discharge, and a necessary adjunct to standard microbiology. Quantitative methods are required to understand species abundance in polymicrobial infections and may be needed to measure accurately the microbiological impact of interventions and to provide a better understanding of clinical failure in these children.

## Background

Indigenous children in remote communities of Australia’s Northern Territory (NT) experience frequent episodes of acute otitis media (AOM) [1]. Bulging of the tympanic membrane caused by suppuration in the middle ear will often lead to spontaneous perforation in this population. A 2001 survey of 709 Indigenous children aged 6 to 30 months reported a 24% prevalence of tympanic membrane perforation (TMP); 7% AOM with perforation (AOMwiP), 2% dry perforation, and 15% chronic suppurative otitis media (CSOM) [2]. Surveys conducted after introduction of pneumococcal conjugate vaccine in the NT have shown <10% of Indigenous children have bilateral normal healthy ears and approximately 20% of children have TMP (unpublished). Randomised controlled trials confirm that otitis media in this population is difficult to cure [1,3,4]. A better understanding of otitis media pathogenesis is needed to guide improved prevention and treatment strategies, particularly in high-risk populations.

Internationally, a compilation of eight studies involving tympanocentesis and culture of middle ear fluid in AOM without perforation reported *Streptococcus pneumoniae,* followed by nontypeable *Haemophilus influenzae* (NTHi), and *Moraxella catarrhalis* as the most commonly cultured bacteria [5]. In studies of the bacteriology of spontaneous otorrhea associated with AOM, *H. influenzae, S. pneumoniae*, and *Streptococcus pyogenes* were primarily identified by culture [6-8]. Tympanocentesis has not been ethically approved for research purposes in the NT, thus only the bacteriology of spontaneous otorrhea associated with AOMwiP and CSOM have been described in NT Indigenous children. Our previous culture-based studies of AOMwiP found NTHi in 55 to 60%, *S. pneumoniae* in 30 to 40%, and *M. catarrhalis* in less than 10% of ear discharge swabs [9]. In children with CSOM, secondary pathogens such as *Pseudomonas aeruginosa* and *Staphylococcus aureus* are more often involved, but NTHi remains a commonly cultured otopathogen [4].

It is unclear whether otitis media is dominated by a single species or whether an array of microbes contribute to disease severity and chronicity. Culture-based studies report co-infection with multiple otopathogens and culture-independent studies have demonstrated a complex polymicrobial middle ear bacteriology [10-12]. The aims of the current study were : i) to measure the prevalence and abundance of *H. influenzae*, *S. pneumoniae* and *M. catarrhalis* in ear discharge from children with AOMwiP to determine if a single species dominates; and ii) to determine if bacterial prevalence and abundance in nasopharyngeal swabs reflects that in the middle ear during AOMwiP.

## Methods

### Ethical approval

Ethical approval for this study was granted by the Human Research Ethics Committee of the Northern Territory Department of Health and Menzies School of Health Research (HREC07/85). The swabs were collected as part of several clinical studies with written consent from a parent/guardian using a consent process (consent form and information sheet) approved by the Human Research Ethics Committee.

### Ear examinations

Ears were examined using video otoscopy and tympanometry. AOMwiP was defined as a perforation for less than 6 weeks’ duration and a perforation size <2% of the pars tensa [3]. Ear discharge swabs were collected following cleaning of debris from the external canal then by positioning swabs as close to the perforation as possible to collect fresh otorrhea at the perforation [13].

### Participants and sample collection

This study analysed 55 paired nasopharyngeal and ear discharge swabs from 51 Indigenous children resident in remote communities of the Northern Territory of Australia. The children were 3.5 – 45.6 months of age. Each swab had been stored in 1.0 ml skim milk-tryptone-glucose-glycerol-broth [14] at -80°C. Swabs were selected from three completed studies: a randomised controlled trial (RCT) of long-term amoxycillin (up to 6 months) versus placebo for treatment of OME in 3 remote Indigenous communities [15]; a longitudinal carriage study of infants receiving 7-valent pneumococcal conjugate vaccine and 23-valent pneumococcal polysaccharide vaccine in three remote Indigenous communities [16]; and an RCT of azithromycin versus amoxicillin for AOM in 16 communities [1]. All the available paired nasopharyngeal and ear discharge swabs were selected provided that the child had not received antimicrobial therapy in the previous seven days, and the parent/guardian had provided consent for further research (where applicable). Where a child had bilateral perforations, the left ear discharge was selected.

### Microbiological analysis and quantitative PCR estimates

10 μl of the original swab medium was cultured using methods optimized for recovery of NTHi, *S. pneumoniae* and *M. catarrhalis*. NTHi were identified from bacitracin-vancomycin-clindamycin chocolate agar plates based on colonial morphology (greyish, semi-opaque, smooth, flat or convex, 1-3 mm in size), X and V growth factor dependence, and lack of reaction with capsular antisera using the Phadebact® Haemophilus coagglutination test. Where recovery of NTHi was compromised by swarming *Proteus* species, a filtration step was included [4]. *S. pneumoniae* were identified from colistin naladixic acid plates based on colonial morphology (flat, usually dimpled, 1-3 mm in size), α-haemolysis, sensitivity to optochin, and the Phadebact® Pneumococcus Test. *M. catarrhalis* were identified from chocolate agar plates based on colonial morphology (discrete, smooth, glistening, white-grey, 1-3 mm colonies), 'hockey puck’ movement when pushed, oxidase production, and Gram stain.

DNA was extracted from 200 μl of each swab as described previously [17]. *H. influenzae, S. pneumoniae, M. catarrhalis*, and total bacterial load (16S rRNA gene amplicon) were estimated by quantitative real-time PCR (qPCR) using methods and acceptance criteria described previously [13]. For *H. influenzae*, the hpd#3 qPCR assay [18] which demonstrated superior discrimination of *H. influenzae* and *H. haemolyticus*[19], was used. Total bacterial load data are estimates which need to be interpreted cautiously due to the biases inherent to the method. Bacteria have varied copy numbers of ribosomal operons (*S. pneumoniae* has 4 copies, and *H. influenzae* contains 7 copies); our assay uses *S. pneumoniae* to create standard curves for quantification. The limit of detection for each qPCR assay was equivalent to 9×10^4^ cells/swab for the total bacterial load assay; 1×10^4^ cells/swab for the *H. influenzae* and *M. catarrhalis* assays; and 0.9×10^4^ cells/swab for the *S. pneumoniae* assay. The limit of quantification was 1×10^5^ cells/swab for the *H. influenzae* and *M. catarrhalis* qPCR assays, and 0.9×10^5^ cells/swab for *S. pneumoniae* and total bacterial load assays. The limit of detection for culture (based on analysis of 10 μl of 1 ml swab) was 1×10^2^ cells/swab.

### Statistical analysis

The bacterial abundance dataset contained a large proportion of zero counts and was not normally distributed. Therefore the non-parametric Wilcoxon matched-pairs signed-ranks test was used to compare bacterial abundance estimates in the paired nasopharyngeal and ear discharge swabs for all data, and for swabs where the ear discharge was positive for the bacterium of interest. All statistical analyses were undertaken using STATA version 12.0 (StataCorp LP). Non-metric multi-dimensional scaling (NMDS) was performed using Primer-E Ltd (v 6.1.13, Plymouth UK) and was based on a Bray-Curtis similarity matrix of 4th root transformed abundance data. The location of each data point in low-dimensional space is based on qPCR estimates of *H. influenzae*, *S. pneumoniae* and *M. catarrhalis*; all swabs negative for the three species were excluded (five swabs). Each data point refers to an ear discharge swab and is coloured according to a dichotomous abundance measure (>10^5^ or <10^5^ cells/ml). A nominal cutoff of 10^5^ cells/ml was selected to represent high versus low or no infection based on other studies [17,20].

## Results

### Detection of pathogens by culture and qPCR

**Figure 1:**
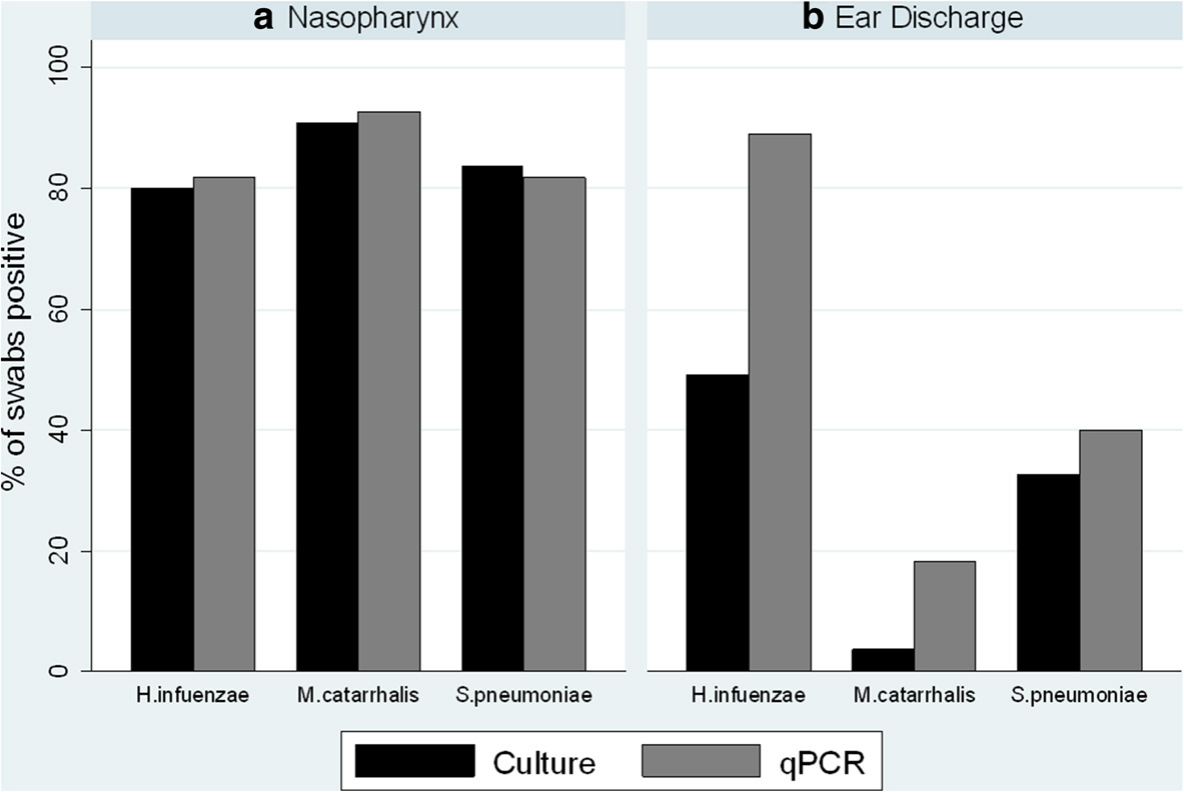
**Culture and qPCR of nasopharyngeal and ear discharge swabs from 55 Indigenous Australian children with AOMwiP.** Percentage of **(a)** nasopharyngeal swabs and **(b)** ear discharge swabs positive by culture and qPCR for *H. influenzae*, *S. pneumoniae* and *M. catarrhalis*.

Among the 55 nasopharyngeal swabs, *H. influenzae*, *S. pneumoniae* and *M. catarrhalis* were detected in 80%, 84%, and 91% by culture and in 82%, 82% and 93% by qPCR. Among the 55 ear discharge swabs, *H. influenzae*, *S. pneumoniae* and *M. catarrhalis* were detected in 49%, 33%, and 3.6% by culture and in 89%, 41% and 18% by qPCR (Figure 1). By qPCR, all three species were detected in 73% (40) of nasopharyngeal swabs, and in 5% (3) of ear discharge swabs; at least two species were detected in 87% (48) of nasopharyngeal swabs and 51% (28) of ear discharge swabs. The sensitivity of the qPCRs was over 80% compared to culture in all cases except for *M. catarrhalis* ear discharge where there were only 2 culture-positive swabs limiting the resolution of such a comparison. The specificities were less consistent due to the relatively small number of swabs that were not positive by both qPCR and culture; with *H. influenzae* the notable exception where 22/28 (79%) of the culture-negative swabs were qPCR positive. Sensitivities and specificities are presented in Table 1.

**Table 1:** Number of swabs positive by qPCR and culture, sensitivity and specificity of qPCR compared with culture

	***H. influenzae***		***S. pneumoniae***		***M. catarrhalis***	
	**NP**	**ED**	**NP**	**ED**	**NP**	**ED**
**qPCR + Culture+**	37	27	42	16	49	1
**qPCR + Culture-**	8	22	3	6	2	9
**qPCR-Culture+**	7	0	4	2	1	1
**qPCR-Culture-**	3	6	6	31	3	44
**Sensitivity**	0.84	1	0.91	0.89	0.96	0.5
**Specificity**	0.27	0.21	0.67	0.84	0.9	0.83

NP, nasopharynx.

ED, ear discharge.

Other potential otopathogens were cultured less frequently; for example, beta-haemolytic streptococci were detected in 5 (9%) of ear discharge swabs by culture, and not in any nasopharyngeal swabs.

### qPCR estimation of bacterial abundance

**Figure 2:**
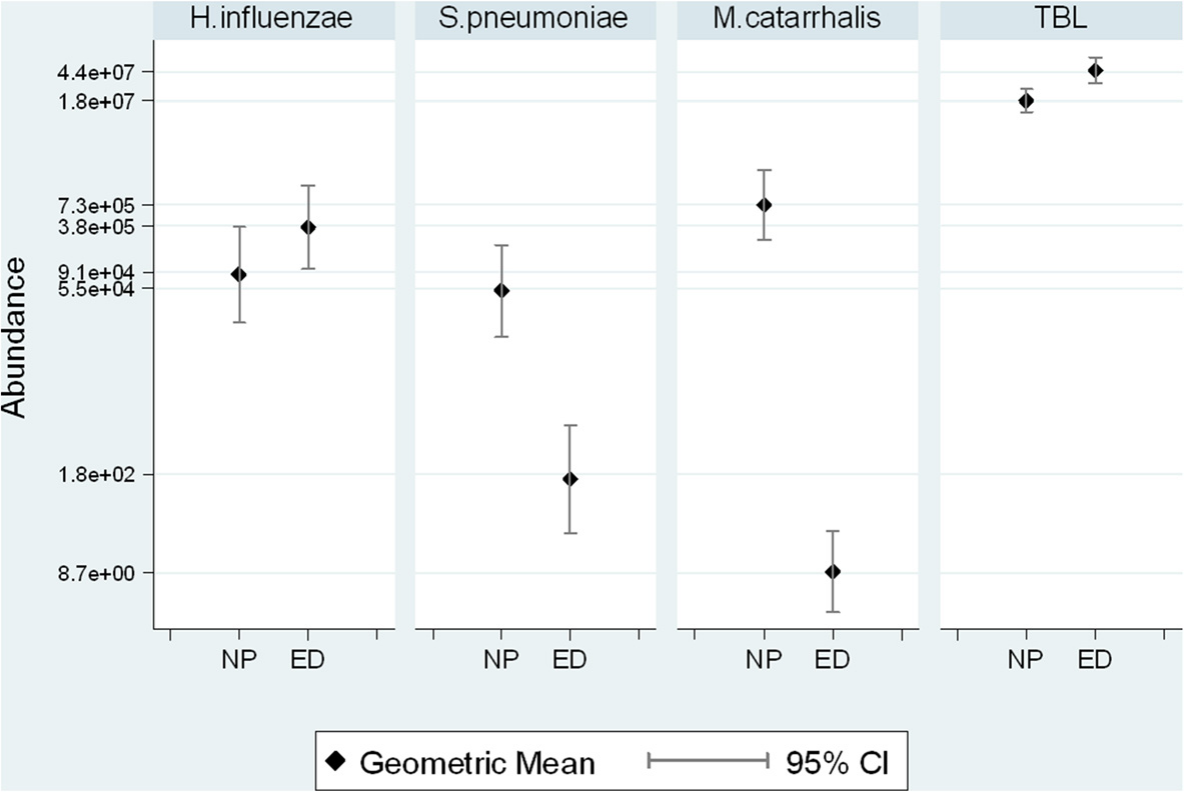
**Bacterial abundance determined by qPCR in nasopharyngeal and ear discharge swabs.** Geometric mean abundance of *H. influenzae*, *S. pneumoniae*, *M. catarrhalis* and total bacterial load (TBL) in nasopharyngeal (NP) and ear discharge (ED) swabs.

The geometric mean bacterial abundance estimates for individual species and for total bacterial load are shown in Figure 2. Highest nasopharyngeal geometric mean bacterial abundance was observed for *M. catarrhalis* (7.3×10^5^ cells/ml, 95% CI 2.5×10^5^-2.1×10^6^), followed by *H. influenzae* (8.5×10^4^ cells/ml, 95% CI 1.9×10^4^-3.7×10^5^) and *S. pneumoniae* (5.2×10^4^ cells/ml, 95% CI 1.3×10^4^-2.1×10^5^). In ear discharge swabs, *H. influenzae* demonstrated the highest geometric mean bacterial abundance (3.6×10^5^ cells/ml, 95% CI 1×10^5^-1.3×10^6^), followed by *S. pneumoniae* (1.6×10^2^ cells/ml, 95% CI 30–8.3×10^2^) and *M. catarrhalis* (9 cells/ml, 95% CI 2.6-31). The large number of *M. catarrhalis* and *S. pneumoniae* negative ear discharge swabs contributed to their low geometric mean abundance. The geometric mean total bacterial loads estimated on a 16S rRNA gene amplicon were 1.8×10^7^ cells/ml (95% CI 3×10^7^-6.8×10^7^) for nasopharyngeal swabs and 4.6×10^7^ cells /ml (95% CI 3×10^7^-6.8×10^7^) for ear discharge swabs.

### Comparison of bacterial abundance in nasopharyngeal and ear discharge swabs

**Figure 3:**
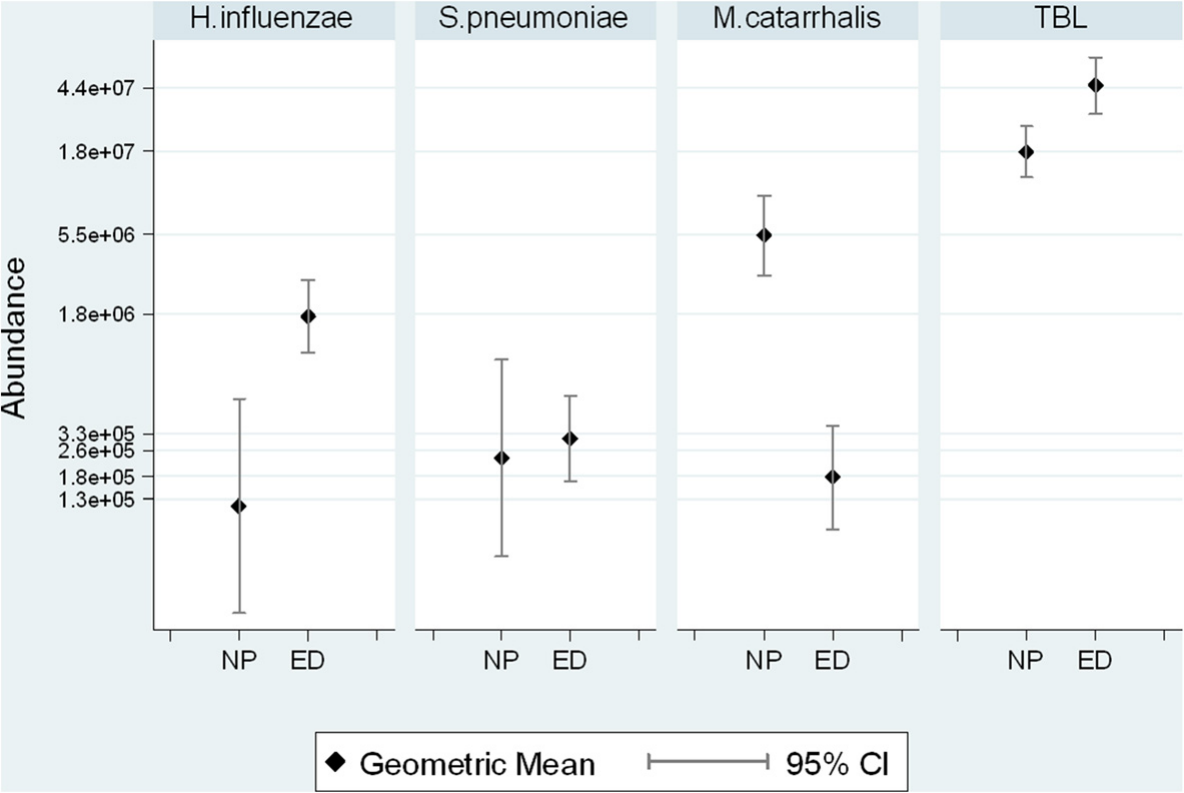
**Bacterial abundance determined by qPCR in paired swabs where ear discharge was positive.** Comparison of qPCR estimates where ear discharge (ED) swabs were positive for the pathogen of interest, and paired nasopharyngeal (NP) swabs. TBL, total bacterial load.

In a comparison of all samples (Figure 2), total bacterial load was significantly higher in ear discharge compared with nasopharyngeal swabs (p = 0.002). Abundance of *S. pneumoniae* (p = 0.001) and *M. catarrhalis* (p < 0.001) was significantly lower in ear discharge, and no significant difference was seen for *H. influenzae* between sites. While these data illustrate the average difference in abundance between these specimen types, the effect of the negative swabs may hide relationships that are dependent on presence of the pathogen. Thus, an analysis of the abundance of each species in qPCR-positive ear discharge swabs and their paired nasopharyngeal swabs was done (Figure 3). With negative qPCR swabs excluded, the geometric mean abundance in ear discharge compared to nasopharyngeal swabs was: significantly higher for *H. influenzae* (p = 0.040) and total bacterial load (p = 0.002); not different for *S. pneumoniae*; and significantly lower for *M. catarrhalis* (P = 0.005; based on only 10 qPCR positive ear discharge swabs).

### Relative abundance of each species as a proportion of the total bacterial load

**Figure 4:**
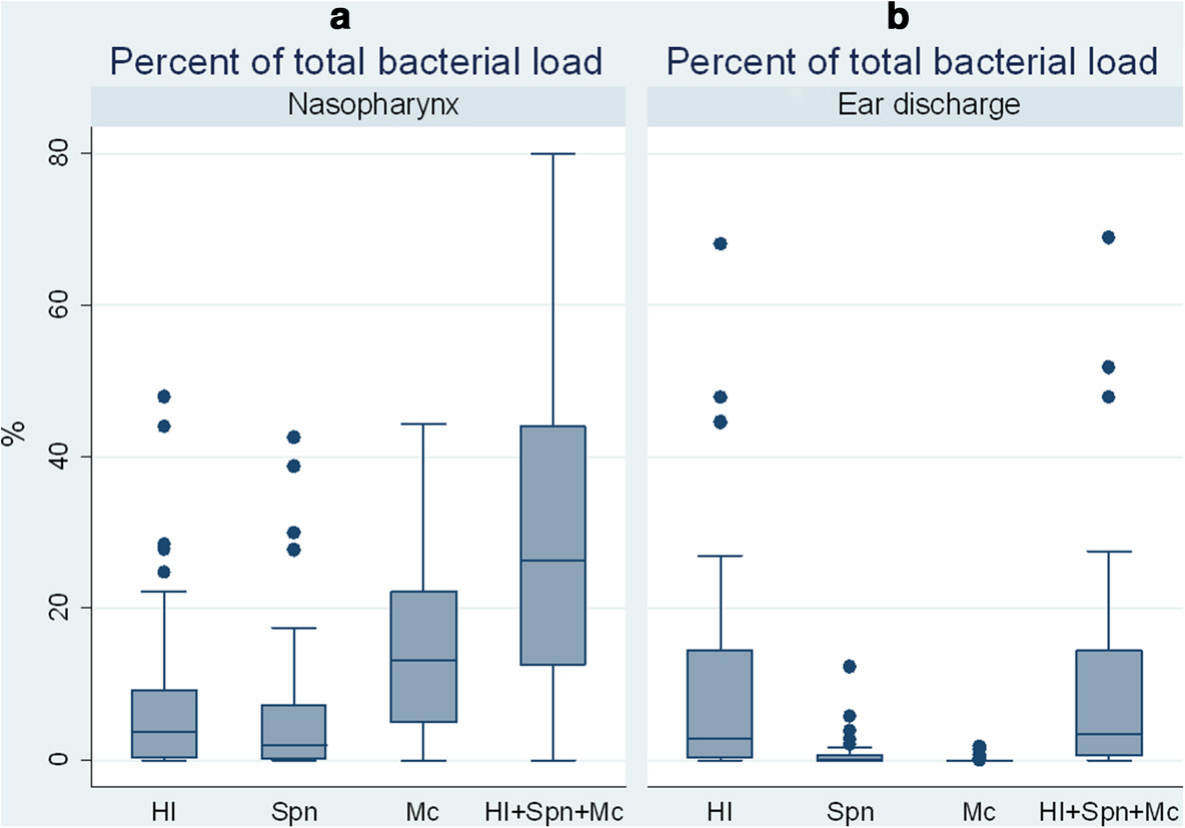
**Relative abundance of *H. influenzae, S. pneumoniae *and *M. catarrhalis *in nasopharyngeal and ear discharge swabs.** Median relative abundance and interquartile ranges for *H. influenzae, S. pneumoniae, M. catarrhalis* and a combination of the three bacteria as a proportion of total bacterial load in nasopharyngeal **(a)** and ear discharge **(b)** swabs.

The relative abundances of individual species were estimated as a proportion of the total bacterial load. The median relative abundances of *H. influenzae*, *S. pneumoniae* and *M. catarrhalis* in nasopharyngeal swabs was 3.8% (range 0-48%), 2% (0-43%) and 13% (0-44%), respectively. In contrast, the median relative abundance of *S. pneumoniae* (0%; range, 0-12%) and *M. catarrhalis* (0%, range 0–1.8%) in ear discharge was low. The median *H. influenzae* abundance in ear discharge was marginally higher at 2.8%, but with a range of up to 68% of the total bacterial load. Figure 4 illustrates the median proportions of *H. influenzae, S. pneumoniae* and *M. catarrhalis* in nasopharyngeal and ear discharge swabs demonstrating the dominance of *H. influenzae* in ear discharge. Notably, a large proportion of the total bacterial load represented bacteria that we did not identify, and which may have originated from the middle ear or the ear canal.

### Non-metric Multidimensional Scaling (NMDS) to visualize the similarity of samples based on bacterial abundance in individual ear discharge swabs

**Figure 5:**
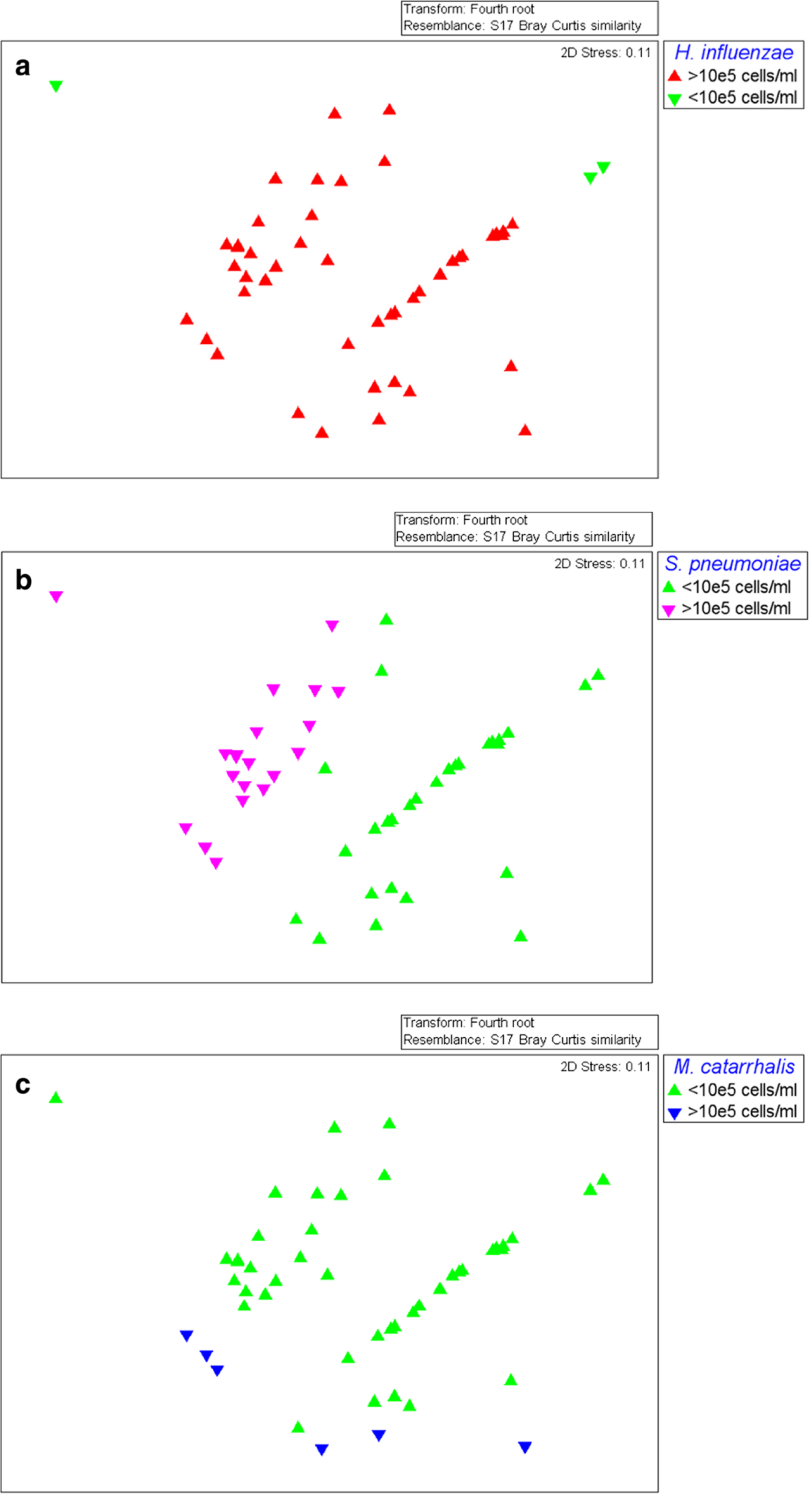
**NMDS visualization of bacterial abundance determined by qPCR in ear discharge swabs.** Non-Metric Multidimensional Scaling (NMDS) for visualization of the similarity of samples based on bacterial abundance in individual ear discharge swabs. Data points are coloured according to a dichotomous abundance measure of >10^5^ cells/ml or <10^5^ cells/ml for **a) ***H. influenzae*; **b) ***S. pneumoniae* and **c) ***M. catarrhalis*. **a)** Data points coloured according to *H. influenzae* abundance; green (<10^5^ cells/ml) or red (>10^5^ cells/ml). **b)** Data points coloured according to *S. pneumoniae* abundance; green (<10^5^ cells/ml) or pink (>10^5^ cells/ml). **c)** Data points coloured according to *M. catarrhalis* abundance; green (<10^5^ cells/ml) or blue (>10^5^ cells/ml).

Conceptualisation of bacterial abundance data from individual swabs is complicated by data from the three variables (*H. influenzae, S. pneumoniae*, *M. catarrhalis* abundance), the large variance in bacterial abundance estimates, and the high frequency of negative results. In this case, we used non-metric multi-dimensional scaling (NMDS) as a tool for visualization of a similarity matrix of individual swabs based on the *H. influenzae, S. pneumoniae* and *M. catarrhalis* abundance data*.* The NMDS uses the similarity matrix to determine the location of each data point (indicative of each swab) in two-dimensional space with similar swabs “clustering” together. As shown in Figure 5a, the majority of ear discharge swabs contained >10^5^ *H. influenzae* cells/ml. Figure 5b and 5c show that ear discharge swabs with >10^5^ *S. pneumoniae* cells/ml or >10^5^ *M. catarrhalis* cells/ml co-exist with *H. influenzae* counts of >10^5^ cells/ml, with a single exception in which *S. pneumoniae* dominated the other species.

## Discussion

In nasopharyngeal swabs collected from Indigenous children with AOMwiP, prevalence of *H. influenzae*, *S. pneumoniae* and *M. catarrhalis* by qPCR was high (82-93%), with 87% of swabs positive for at least two pathogenic species. In paired ear discharge swabs, *H. influenzae* was not only a very commonly detected pathogen (89% of swabs by qPCR), it was more dominant, in terms of abundance, than *S. pneumoniae* and *M. catarrhalis*. *S. pneumoniae* was next most common and abundant in ear discharge, while *M. catarrhalis* (the dominant otopathogen in the nasopharynx) had low prevalence and abundance in ear discharge. At least two otopathogenic species were detected concurrently in 51% of ear discharge swabs; in addition, beta-haemolytic streptococci were detected in 9% of ear discharge swabs by culture, and may require further investigation in future studies. The contribution of various otopathogens to polymicrobial ear disease remains to be determined, and is likely to change during different stages of infection.

Estimation of the total bacterial load in swabs made possible the estimation of the relative abundance of the pathogens in each specimen. While *H. influenzae*, *S. pneumoniae* and *M. catarrhalis* generally represent a small proportion of the total bacterial load in ear discharge, *H. influenzae* could reach proportions of 68% and was clearly the dominant pathogen amongst these three otopathogens. Visualisation of the individual swabs using NMDS supported the primary statistical analysis that *H. influenzae* frequently exceeds abundance measures of 10^5^ cells/ml, and where ear discharge swabs contained *S. pneumoniae* or *M. catarrhalis* at abundance measures greater than 10^5^ cells/ml, this was always in the presence of high *H. influenzae* abundance (>10^5^ cells/ml) with one exception where *S. pneumoniae* dominated. Numerous studies have demonstrated the importance of *H. influenzae*, *S. pneumoniae* and *M. catarrhalis* in otitis media; however, the generally minor representation of these three species among the total bacterial load of most ear discharge swabs highlights the need for future investigations of AOMwiP bacteriology using complementary qPCR and microbiomic analyses.

*H. influenzae* is also a dominant pathogen in chronic lower respiratory disease in this population. Infection of the lower airways with NTHi (defined as >10^4^ colony forming units per ml bronchoalveolar lavage fluid) was identified in 47% of Australian Indigenous children with non-cystic fibrosis bronchiectasis, compared with an 18% prevalence of *S. pneumoniae* lower airway infection [21]. The involvement of NTHi in mucosal diseases likely reflects a balance between high carriage rates, its status as an opportunistic pathogen, its ability to persist in a polymicrobial environment, and the host immune response. In its planktonic form, NTHi is effectively killed by complement [22] and serum antibodies [22,23] and is not commonly associated with systemic disease. Infection of the respiratory mucosa is likely reliant on a complex interaction of pathogen and host mucosal factors with data from adults with chronic lower respiratory disease implicating the importance of cell mediated immunity [24].

The discrepancy between culture and molecular detection techniques in ear discharge swabs was marked and clearly demonstrates that PCR is a necessary adjunct to standard microbiology for this specimen type. Biofilm [25] and intracellular infection [25,26] which are proposed mechanisms employed by NTHi to survive and persist in the hostile mucosal environment represent one explanation for the discrepancy*.* Other explanations for the qPCR-positive, culture-negative swabs may be related to non-viable cells, an altered metabolic state [27] of the bacteria in the middle ear, or other determinants as discussed in greater detail elsewhere [17]. The culture-positive, qPCR-negative swabs cultured <10 colonies and were below the limit of detection for qPCR (as detailed in Methods), with the exception of two swabs which may have experienced PCR inhibition. For the qPCR-positive, culture-negative swabs, the bacterial loads varied widely.

A limitation of this study was the likely variation in quantity of swab material collected at the two sites and between individuals; however, our analysis of relative abundance, which provides a within sample measure, did not change the results. Collection of middle ear effusion from Indigenous Australian children by tympanocentesis for research purposes has not been ethically approved; thus our study relied on examination of otorrhea following spontaneous perforation. Despite our protocols to collect fresh otorrhea at the perforation following cleaning of the ear canal, these swabs may include canal flora (for example, *P. aeruginosa*), which may or may not be contributing to the infection. A further potential bias relates to the effect of the numerous other taxa present but unidentified in each swab, and originating from the middle ear or the ear canal, which may influence the abundance or possible interdependencies of the three main otopathogens. Finally, our study does not address the question of whether abundance of a pathogen in a swab is related to its contribution to infection; for the three otopathogens some support is provided by findings that nasopharyngeal load of these otopathogens was significantly associated with presence and severity of current ear disease [17,28].

## Conclusions

In conclusion, *H. influenzae* was the most prevalent and abundant pathogen identified in middle ear discharge from Indigenous children with AOMwiP. In the case of *S. pneumoniae* and *M. catarrhalis*, high prevalence and abundance in the nasopharynx did not predict ear discharge microbiology. qPCR was substantially more sensitive than culture for ear discharge, and a necessary adjunct to standard microbiology. Quantitative methods are required to measure species abundance and relative abundance in polymicrobial infections and better understand pathogenesis. Such methods may be needed to accurately measure the effectiveness of treatment and prevention interventions for complex polymicrobial infections such as otitis media and respiratory tract infections.

## Competing interests

The authors declare that they have no competing interests.

## Authors’ contributions

HSV conceived the study, obtained funding and ethical approvals, and drafted the manuscript; MJB, RLM and KMH generated the data; MJB, MK and LW analysed the data; SJP, RLM, and RBT helped to draft the manuscript; PSM and AJL advised on intellectual content and provided critical manuscript review. All authors approved the final manuscript. All authors read and approved the final manuscript.

## Pre-publication history

The pre-publication history for this paper can be accessed here:

http://www.biomedcentral.com/1472-6815/13/12/prepub
